# A proper reference metabolic equivalent value to assess physical activity intensity in Japanese female nurses

**DOI:** 10.1186/s40695-019-0048-3

**Published:** 2019-09-13

**Authors:** Yuki Ideno, Kunihiko Hayashi, Jung Su Lee, Yukiko Miyazaki, Shosuke Suzuki

**Affiliations:** 1 Gunma University Initiative for Advanced Research, 3-39-22 Showa-machi, Maebashi City, Gunma 371-8511 Japan; 2 Graduate School of Health Science, Gunma University, 3-39-15 Showa-machi, Maebashi City, Gunma 371-8514 Japan; 3 Graduate School of Medicine, The University of Tokyo, 7-3-1 Hongo, Bunkyo-ku, Tokyo, 113-0033 Japan; 4 Department of Nursing, Gunma Prefectural College of Health Sciences, 323-1 Kamioki-machi, Maebashi, Gunma 371-0052 Japan; 5 Emeritus, Gunma University, 3-39-22 Showa-machi, Maebashi City, Gunma 371-8511 Japan

**Keywords:** Physical activity questionnaire, Accelerometer, Reference metabolic equivalent value, Total energy expenditure, Moderate to vigorous intensity physical activity

## Abstract

**Background:**

Various questionnaires have been developed to assess physical activity, but only a few simple questionnaires are suitable for self-administration in large groups of midlife working women. This study examined the usefulness of the Japan Nurses’ Health Study (JNHS) questionnaire for self-administered physical activity surveys.

**Methods:**

The JNHS physical activity questionnaire consisted of items covering seven degrees of intensity. The metabolic equivalents (METs) for the physical activity intensity of the questionnaire were estimated from energy expenditure as measured by a uniaxial accelerometer with the Markov Chain Monte Carlo (MCMC) simulation. The estimated METs were then assigned to the JNHS baseline survey data, and the total energy expenditure (TEE) and the time spent performing ≥3 METs hour of physical activity, called moderate to vigorous intensity physical activity (MVPA), were calculated.

**Results:**

For working situations, application of the MCMC simulation resulted in estimated reference values of 1.2 METs for “sitting work”, 1.6 METs for “standing work”, 1.8 METs for “walking work”, and 4.5 METs for “heavy work”. For non-working situations, the estimated values were 1.1 METs for sedentary time, 2.4 METs for “moderate physical activity”, 4.4 METs for “vigorous physical activity”, and 9.4 METs for “very vigorous physical activity”. When these estimated METs were used, the mean TEE/day was 1808 kcal. This corresponded to − 3.0% of the TEE/day generated by the accelerometer. These estimated MET values showed similar results as a previous study measuring activity using the doubly-labeled water method. The number of hours per week of MVPA significantly decreased with age, which is also consistent with previous findings.

**Conclusions:**

Estimated reference MET values in this study were similar to those in previous studies of Japanese women. The JNHS questionnaire is therefore useful for epidemiological surveys of midlife working women because it assigns estimated MET values as physical activity intensities.

## Background

Measures for obesity and associated diseases, such as diabetes, hypertension, and hypercholesterolemia, have become an important issue in Japan in recent years. Numerous studies have shown that regular physical activity reduces the risk of these diseases in adults, and relationships between physical activity and cardiovascular disease, stroke, and diabetes have been shown [1–3].

Physical activity guidelines state that it is performant for health to do at least 150 min per week of moderate-to-vigorous physical activity (MVPA), equivalent to at least three metabolic equivalents (METs) [4, 5]. The Centers for Disease Control and Prevention and the American College of Sports Medicine guidelines recommend that people “accumulate at least 30 min/day of MVPA, in either one continuous bout or several shorter bouts lasting 8–10 min, on 5 days or more per week” [4]. In Japan, the “Exercise and Physical Activity Guide for Health Promotion in 2006” [5] states that people should do physical activity at this intensity to improve health, and states “Let’s target 23 Exercise (Ex) (Ex = MET × hour, i.e. unit of quantity) per week by physical activity, of which 4 Ex is by active exercise (≥4 METs)!”

At present, most of the evidence on physical activity is based on studies from Western countries. Epidemiological studies of midlife women are needed to identify the intensity, frequency, and quantity of physical activity required to help to avoid various health conditions. It is therefore important to develop simple assessment methods for physical activity that can be used in large epidemiological studies. However, when targeting a large population, objective measurement methods like using a human calorimeter or an accelerometer, or the doubly-labeled water (DLW) method, are impractical because of the cost, and the burden they place on participants. Various attempts have therefore been made to create a self-administered physical activity questionnaire [6, 7]. Examples include the International Physical Activity Questionnaire (IPAQ), which is versatile and used internationally [7].

There are two forms of the IPAQ. The long version (31 questions) was designed to collect detailed information within five domains. The short version (nine questions) has no separate domains. The Japan Nurses’ Health Study (JNHS) is a comprehensive epidemiological study of the overall lifestyle of female nurses [8]. Detailed investigations, such as the IPAQ long version, are difficult to use in large-scale epidemiological surveys. Working women’s mental and physical health is also considered to be influenced by their working conditions, including levels of physical activity. This makes the IPAQ short version, which does not distinguish between working and non-working hours, unsuitable for the JNHS population. Both simplification and distinction of working and non-working hours was required.

In considering prevention of disease and promotion of health, it is important to examine the relationship between daily physical activity and energy expenditure (EE). The Compendium of Physical Activities [9] provides a convenient five-digit coding scheme that can be used to classify activities by rate of EE or METs. However, MET values need to be assigned for categories such as moderate or vigorous physical activity. Few studies have investigated the validity of assigned MET values for physical activity questionnaires.

The validity of EE as estimated by physical activity questionnaires has been examined in comparison with objective activity measurement devices, such as DLW and accelerometers. Some studies have shown that the correlation is high, but others have shown that questionnaires may both overestimate and underestimate EE [10, 11]. Neilson et al. [11] found that discrepancies between physical activity questionnaires and DLW estimates may be attributable to inaccurate assignment of METs to self-reported activities. This study therefore aimed to estimate the MET values corresponding to the JNHS question items using an accelerometer and to validate these values.

## Methods

The study was split into two parts. Study 1 aimed to estimate the proper MET values for the physical activity intensity corresponding to the JNHS questionnaire items. EE derived from the JNHS self-administered questionnaire was quantified in a subsample of JNHS (Gunma Nurses’ Health Study [GNHS]). The mean METs for physical activity in the pre-specified subcategories (for example, work time and off-duty physical activity time) were put into a Markov Chain Monte Carlo (MCMC) model. A second estimate of EE derived from uniaxial accelerometer measures was used as a reference. The mean difference and percentage difference between those two estimates were used to evaluate accuracy.

Study 2 tested the validity of the estimates of total energy expenditure (TEE) derived from the JNHS questionnaire. These estimates were examined for association with age groups using the whole baseline JNHS sample. The TEE per day was calculated from the estimated MET values from Study 1 or the middle value of the intensity range (midrange) MET values. These data were then compared for five age groups.

### Study population

The JNHS surveyed 48,618 female nurses, aged 25 to 69 years, who participated in the 2001–2007 nationwide baseline survey. Participants were excluded from the data analyses for the following reasons: pregnancy (*n* = 998); missing values for weight or height (*n* = 1416); or missing values for the questions about physical activities (*n* = 15,267). A total of 30,937 participants were included in the analyses for Study 2.

A pilot study (GNHS) among 698 nurses was conducted before the main JNHS. From these 698 nurses, 41 women agreed to participate in our Study 1 and provided written informed consent.

The research was conducted in two periods (summer to fall and winter to spring). Participants were excluded from the data analyses for failure to maintain a sleep diary (*n* = 2), and having forgotten to wear the accelerometer (*n* = 7), except when sleeping. Participants were also excluded if they had missing values for the questions about physical activities (*n* = 1). The final number that was analyzed was 31 participants. For those who participated in both seasons (*n* = 9), the mean of the two seasons was used for analysis, because the JNHS self-administered questionnaire asked how much time in total participants spent on each of the activities ‘on average per week, during the past year’.

### Survey methods

The validity study was carried out in August–October 2007 and February–April 2008. The participants were surveyed on their lifestyles, including a sleep survey [12], and a survey of physical activities of daily living. To investigate the usual physical activity per day, the survey period was 7 consecutive days including holiday(s).

Participants who had participated in the GNHS were mailed a letter requesting their cooperation and participation in this study. Prior informed consent was obtained from each participant, and they were given the questionnaire, accelerometer, and a sleep diary. Physical measurements, including height and weight, were made at that time. The questionnaire, accelerometer, and sleep diary were recovered through the postal system upon completion of the survey.

#### Sleep time

For 7 consecutive days, each participant recorded the following data in her sleep diary: the time she went to bed, the time she got out of bed, and the duration of any naps. The sleeping hours for each participant were then calculated from the diary.

#### Physical activity

The self-administered questionnaire contained questions about the time spent doing physical activities during 1 week. Three subcategories were set for physical activity during off-duty hours: “moderate”, “vigorous”, and “very vigorous” physical activities. Physical activity examples of 3.0–5.0, 5.0–8.0, and over 8.0 METs were provided for the three subcategories. Four subcategories were set for on-duty physical activity: “sitting”, “standing”, “walking”, and “heavy work (such as transportation of heavy objects)”. Physical activity examples of 1.0–1.5, 1.2–2.0, 2.0–4.0, and 4.0–8.0 METs were provided for the four subcategories. A midrange value of the METs was assigned to each subcategory. For off-duty physical activity/sports, 3.0 METs were assigned for “moderate”, 6.0 for “vigorous”, and 10.0 for “very vigorous” physical activity. The remaining time, excluding sleep time, was defined as off-duty light physical activity, and given a MET of 1.5. For on-duty physical activity, values were assigned of 1.3 METs for “sitting”, 1.5 for “standing”, 2.5 for “walking”, and 6.0 for “heavy work”.

Both the validity study and the JNHS baseline survey used the same questionnaire.

#### Lifecorder energy expenditure

To evaluate EE, the study used a uniaxial accelerometer called the Lifecorder (Kenz Lifecorder EX, Suzuken Co., Ltd., Nagoya, Japan). The participants were asked to attach the Lifecorder to their waist for 7 consecutive days (24 h × 7 days). This device was not waterproof, so participants were asked to record the time and type of physical activity when the device was removed or not attached. When the Lifecorder was not attached, a physical activity intensity was assigned using the Compendium of Physical Activities [9] when the activity type was clear, and its EE was calculated. The EE for sleep time was calculated as the basal metabolic rate (BMR) × (sleep time/24 h). For bathing, the EE was calculated as 1.5 METs/h. The TEE value was calculated by adding the EE value generated by the Lifecorder to this imputed value. The equations for calculation of the BMR and the EE for each physical activity are shown below [13, 14].

$$ EE\ (kcal)=\left( METs\times time\ spent\ \left[h\right]\right)\times weight\ (kg)\times 1.05 $$

$$ BMR\ (kcal)= basal\ metabolic\ standard\ Japanese\ value\ \left( kcal/{m}^2/h\right)\times body\ surface\ area\ (BSA)\ \left({cm}^2\right)\times 24\ (h)\times \left(1/10000\right) $$

$$ BSA\ \left({cm}^2\right)={weight}^{0.444}\ (kg)\times {height}^{0.663}\ (cm)\times 88.83 $$

$$ Questionnaire\  TEE=\left( BMR\times sleeping\ time/24\ h\right)+ TEF+{\sum}_{i=1}^8\left( weight\ (kg)\times 1.05\times PAi\times METi\right) $$

#### Questionnaire energy expenditure

The study aimed to estimate usual energy expenditure, defined as long-term average expenditure, using a questionnaire about physical activity in daily life in a large study population. We developed a questionnaire that asked about the average hours per week spent doing a particular intensity of activity in the past year. Each day (24 h) was divided into working time, off-duty physical activity time, off-duty light physical activity time, and sleeping time. The sum of the EE in those time periods was combined with the thermic effect of food (TEF) (kcal), calculated as 1/10 × TEE, and that total was defined as the TEE per day. The TEE/day was also defined as the average value of the TEE measured by the Lifecorder during the 7 consecutive days. The sleeping time per day was the average value across the 7-day sleep diary. The working time (PA_1_, “sitting”; PA_2_, “standing”; PA_3_, “walking”; and PA_4_, “heavy work”) and the off-duty physical activity time (PA_5_, “moderate”; PA_6_, “vigorous”; and PA_7_, “very vigorous” physical activity) were obtained from the responses to the questionnaire. The remaining time was defined as off-duty light physical activity time (PA_8_).

$$ \mathrm{Questionnaire}\ \mathrm{TEE}=\left(\mathrm{BMR}\times \mathrm{sleeping}\ \mathrm{time}/24\ \mathrm{h}\right)+\mathrm{TEF}+{\sum}_{i=1}^8\left( weight\ (kg)\times 1.05\times PAi\times METi\right) $$

MET*i* is physical activity intensity (MET) for PA*i*.

1.0 ≤ MET_1_ < 1.5, 1.2 ≤ MET_2_ < 2.0, 2.0 ≤ MET_3_ < 4.0, 4.0 ≤ MET_4_ < 8.0, 3.0 ≤ MET_5_ < 5.0, 5.0 ≤ MET_6_ < 8.0, 8.0 ≤ MET_7_, and 1.0 ≤ MET_8_ < 2.0.

### Statistical analysis

The model includes unknown parameters of physical activity intensities (METs) with a truncated skew distribution in eight intensity sub-categories. In such a complicated and restricted situation, general regression programs failed to converge. The study therefore used computational thinking in a Bayesian way via the MCMC simulation algorithms for sampling from a desired probability distribution to solve the problem [15–17]. The MCMC procedure from SAS software [18] was used to estimate the METs corresponding to the physical activity intensities for the self-administered question items and the off-duty light physical activity time (PA_1_–PA_8_). Gamma distributions were used as the posterior distributions. All of the posterior summary statistics and parameter estimates from 10,000 MCMC simulations were carried out after a burn-in period of 1000 iterations. To compare the values for the TEE/day based on the Lifecorder (Lifecorder TEE) and the TEE/day based on the self-administered questionnaire (Questionnaire TEE), the mean difference (Questionnaire TEE − Lifecorder TEE) and percentage difference ([Questionnaire TEE − Lifecorder TEE]/Lifecorder TEE × 100) between them were calculated. Bland–Altman plots and intra-class correlation coefficients (ICCs) were used to show agreement. The Questionnaire TEE was calculated using the estimated METs from the MCMC simulation and compared with the midrange METs.

The TEE/day was calculated from the METs estimated in Study 1 or the midrange METs. These data were compared across five age groups (25–29, 30–39, 40–49, 50–59, and 60–69 years old). The MET for sleeping was defined as 1.0. The time spent per week on physical activity of at least three METs (MVPA) was also calculated, and the change in that value as a function of age was examined. The midrange METs were used for comparison with the estimated METs. One-way ANOVA and post hoc analysis (Tukey’s test) were used to determine any differences between the five age groups and the TEE/day. The Jonckheere–Terpstra test was used to assess trends across age groups in MVPA. A multiple comparison procedure (Mann–Whitney U test with Bonferroni adjustment) was used to determine differences between age groups. A value of *p* < 0.05 was used to be statistically significant. All analyses used SAS ver 9.3 statistical software (SAS Institute, Cary, NC, USA).

## Results

### Study 1

#### Characteristics of the study participants

Table 1 summarizes the characteristics of the study participants. The mean ± standard deviation (SD) age was 48.7 ± 11.0 years and the mean body mass index was 22.4 ± 3.2 kg/m^2^. The mean TEE/day measured by the Lifecorder was 1861 ± 198 kcal.

**Table 1:** Characteristics of study participants of Study 1 (*n* = 31)

	mean (SD)	median (range)
Age (years)	48.7 (11.0)	46.5 (31.0-70.0)
BMI (kg/m^2^)	22.4 (3.2)	21.3 (17.8-30.8)
Total energy expenditure (kcal/day)	1,860.5 (197.9)	1,869.8 (1,470.4-2,242.7)
Hours of sleep (hr/day)	7.2 (0.6)	7.4 (5.6-8.4)
Hours of working (hr/week)	38.5 (13.9)	41.0 (4.0-56.0)
Sitting for working (hr/week)	13.9 (12.9)	10.0 (0.0-50.0)
Standing for working (hr/week)	12.1 (9.9)	10.0 (0.0-45.0)
Walking for working (hr/week)	11.4 (10.6)	10.0 (0.0-40.0)
Heavy work (hr/week)	1.1 (2.2)	0.0 (0.0-10.0)
Light physical activity (min/week)	105.2 (101.7)	70.0 (0.0-420.0)
Moderate physical activity (min/week)	36.1 (72.9)	0.0 (0.0-280.0)
Vigorous physical activity (min/week)	7.4 (24.2)	0.0 (0.0-120.0)

#### Estimation of physical activity intensities (METs)

The MCMC simulation was used to estimate the reference MET values corresponding to the physical activity intensities for the questions in the self-administered JNHS questionnaire on working times and off-duty physical activity time. The estimated values were 1.2 METs for “sitting work” (95% confidence interval [CI]: 0.89–1.51), 1.6 for “standing work” (95% CI: 1.21–2.12), 1.8 for “walking work” (95% CI: 1.16–2.44), and 4.5 for “heavy work” (95% CI: 3.25–5.89). For off-duty activity, 1.1 METs was estimated for “light” activity (95% CI: 1.00–1.28), 2.4 for “moderate” (95% CI: 1.14–4.25), 4.4 for “vigorous” (95% CI: 2.12–7.02), and 9.4 for “very vigorous” (95% CI: 6.33–13.1)".

**Fig. 1:**
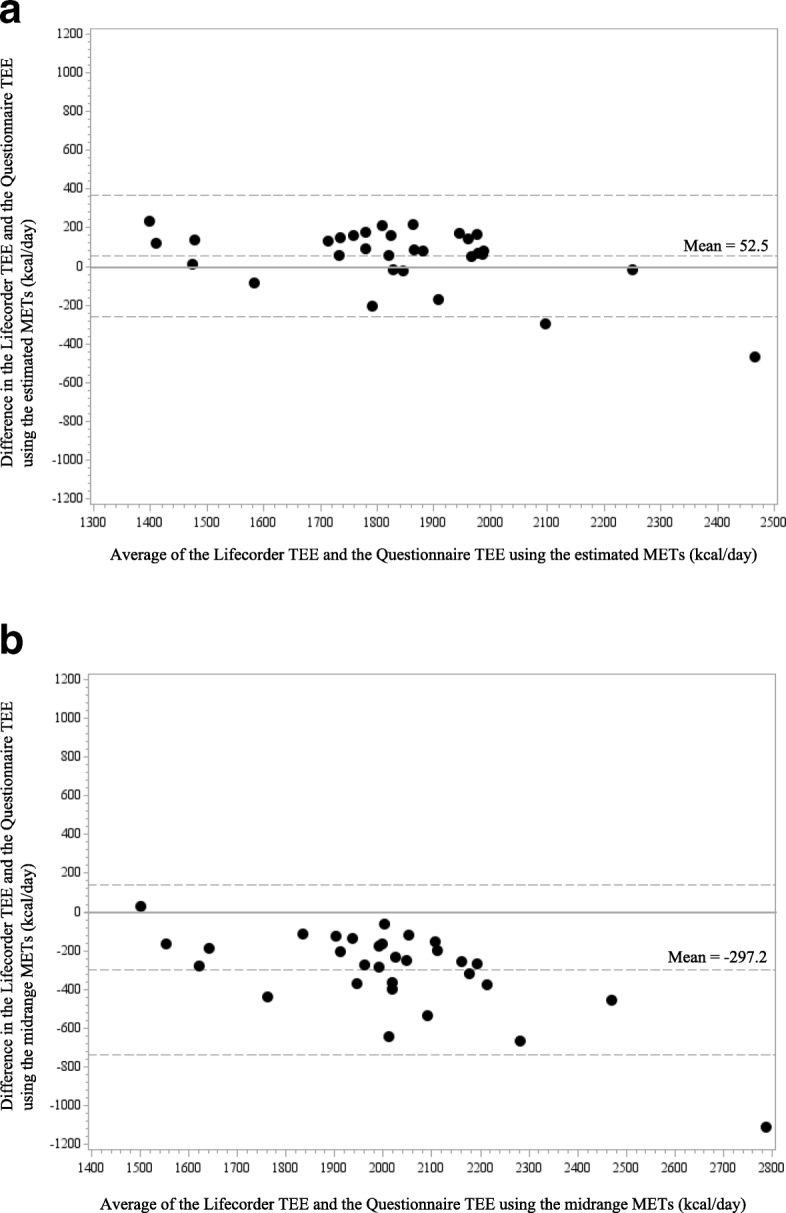
Bland-Altman plot showing differences in the total energy expenditure (TEE)/day based on the Lifecorder and the self-administered questionnaire. **a** Differences in the Lifecorder and the TEE using estimated METs. **b** Differences in the Lifecorder and the TEE using the midrange METs

Using these estimated METs, the mean TEE/day calculated from the responses to the questionnaire was 1808 ± 273 kcal. This value differed from the TEE generated by the Lifecorder by 53 ± 156 kcal. When the midrange METs were used, the Questionnaire TEE/day was 2158 ± 337 kcal. This differed from the Lifecorder TEE by 297 ± 220 kcal. The percentage difference was 3.0%, which was smaller than the 16% found using the midrange METs. Individual results are shown in the Bland-Altman plots in Fig. 1.

Using the estimated METs and midrange METs, the ICCs between the Lifecorder TEE/day and the two different Questionnaire TEEs/day were 0.77 (95% CI: 0.58–0.88) and 0.44 (95% CI: − 0.10–0.76).

### Study 2

#### Characteristics of the study participants

Table 2 shows the characteristics of the study participants. The mean age was 41.3 ± 7.8 years and the mean body mass index was 21.8 ± 2.9 kg/m^2^. The mean sleeping time was 6.5 ± 0.9 h/day and the mean working time was 39.6 ± 13.0 h/week.

**Table 2:** Characteristics of study participants of Study 2 (mean [SD])

	All	25≤ age <30 y	30≤ age <40 y	40 ≤ age <50 y	50 ≤ age <60 y	60 ≤ age <70 y
	(*n* = 30,937)	(*n* = 490)	(*n* = 13,553)	(*n* = 11,547)	(*n* = 5,093)	(*n* = 254)
Age (years)	41.3 (7.8)	27.4 (1.4)	34.5 (2.8)	44.2 (2.9)	53.1 (2.6)	61.8 (2.2)
BMI (kg/m^2^)	21.8 (2.9)	20.3 (2.6)	21.1 (2.8)	22.2 (2.8)	22.8 (2.8)	22.7 (2.8)
Hours of sleep (hr/day)	6.5 (0.9)	6.3 (1.0)	6.5 (1.0)	6.4 (0.9)	6.4 (0.9)	6.5 (0.9)
Hours of working (hr/week)	39.6 (13.0)	39.9 (13.4)	40.3 (12.8)	39.4 (13.1)	38.5 (12.9)	34.0 (13.9)
Sitting for working (hr/week)	8.2 (9.0)	7.9 (8.1)	7.0 (7.4)	8.2 (9.1)	11.0 (11.7)	13.6 (12.8)
Standing for working (hr/week)	17.1 (14.0)	15.9 (13.1)	17.6 (14.0)	17.0 (14.0)	16.1 (14.1)	11.8 (12.9)
Walking for working (hr/week)	12.6 (12.5)	13.7 (12.0)	13.6 (12.6)	12.5 (12.6)	10.3 (11.9)	7.8 (10.6)
Heavy work (hr/week)	1.8 (3.3)	2.5 (3.7)	2.1 (3.5)	1.7 (3.2)	1.2 (2.8)	0.8 (2.6)
Light physical activity (min/week)	113.2 (115.2)	88.9 (86.1)	104.2 (110.4)	115.3 (114.7)	132 (125.8)	161.8 (147.5)
Moderate physical activity (min/week)	21.9 (61.0)	25.1 (59.9)	19.6 (56.0)	20.6 (60.4)	29.5 (71.7)	41.0 (92.6)
Vigorous physical activity (min/week)	2.3 (18.4)	4.3 (23.1)	2.5 (19.1)	1.9 (16.2)	2.7 (20.7)	1.9 (16.5)

#### Total energy expenditure per day

**Fig. 2:**
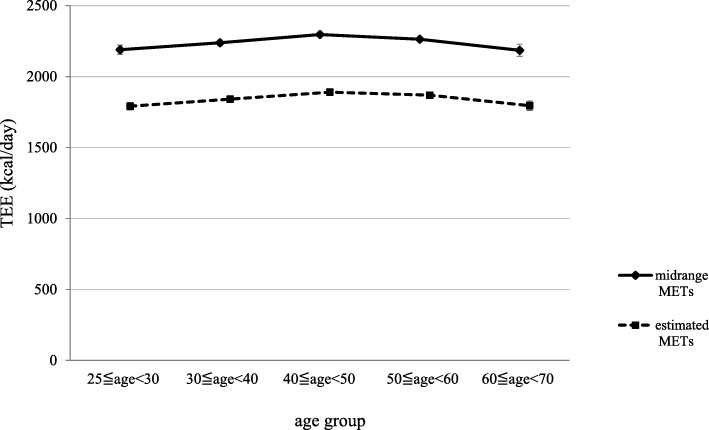
The relationships between age and the total energy expenditure (TEE)

To test the validity of the TEE derived from the JNHS questionnaire in Study 1, which estimated MET values, the association of TEE with age group was examined using the full sample from the JNHS baseline population. Using the estimated METs, the median (interquartile range) and the mean were 1821 (1663–2015) kcal and 1863 ± 292 kcal. Using the midrange METs, the median was 2208 (2006–2457) kcal and the mean was 2262 ± 372 kcal. The means for each age group are shown in Fig. 2. The effect of age was significant (*p* < 0.0001), and significant differences were found in all the age group comparisons except for 20s vs 60s and 30s vs 60s.

#### Time spent on moderate-to-vigorous physical activity

**Fig. 3:**
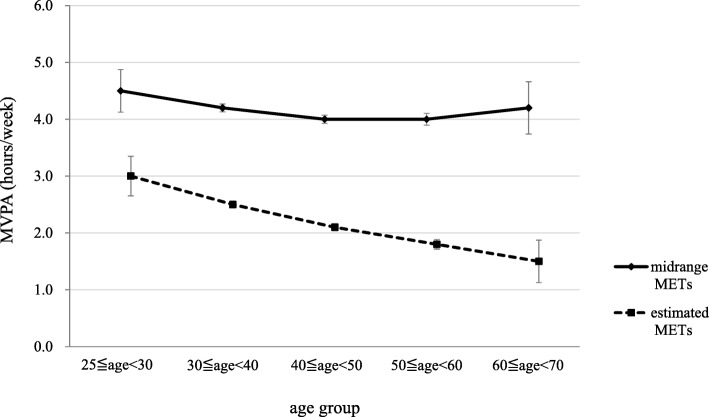
The relationships between age and the time spent doing physical activity expending at least three METs (MVPA)

The time spent MVPA (≥3 METs-h/week) was calculated, and these values were compared between the age groups. When the estimated METs were used, the mean time spent on MVPA for all participants was 2.2 ± 3.5 h/week. The values for age groups were 3.0 ± 3.9 h/week for 25–29 years; 2.5 ± 3.6 h/week for 30–39 years; 2.1 ± 3.4 h/week for 40–49 years; 1.8 ± 3.1 h/week for 50–59 years; and 1.5 ± 3.0 h/week for 60–69 years (Fig. 3). There was a trend towards more time spent doing MVPA when younger (*p* < 0.0001, Jonckheere–Terpstra test). Significant differences were found in all the age groups comparisons except for 50s vs 60s.

Using the midrange METs, the mean time spent on MVPA for all participants was 4.1 ± 4.0 h/week. For each age groups, the values were 4.5 ± 4.2 h/week for 25–29 years; 4.2 ± 4.1 h/week for 30–39 years; 4.0 ± 4.0 h/week for 40–49 years; 4.0 ± 3.8 h/week for 50–59 years; and 4.1 ± 3.7 h/week for 60–69 years (Fig. 3). There was again a trend towards younger people spending more time on this level of activity (*p* < 0.05, Jonckheere–Terpstra test), but there were no significant differences between age groups.

## Discussion

Using simple questions on daily physical activity from the JNHS questionnaire, the METs for different physical activity intensities were estimated by comparing the Questionnaire TEE and the Lifecorder TEE. The estimated values were 1.2 METs for “sitting work”, 1.6 METs for “standing work”, 1.8 METs for “walking work”, 4.5 METs for “heavy work”, 2.4 METs for “moderate physical activity”, 4.4 METs for “vigorous physical activity”, 9.4 METs for “very vigorous physical activity”, and 1.1 METs for “light physical activity” off-duty. The percentage difference between the Lifecorder TEE and the TEE using these estimated METs was 3.0%, which was smaller than the 16% difference using the midrange METs. The correlation coefficient was also larger using the estimated METs than the midrange METs. The Lifecorder tends to underestimate the amount of physical activity, but it is adequate for evaluating the daily amount of physical activity for epidemiological research [19]. We therefore believe that the JNHS question items for physical activity and the estimated MET values are adequate for use in epidemiological research.

Table 3 shows the TEE data obtained from previous studies among Japanese women, with measurements using the DLW method or the human calorimeter. The TEEs calculated from the estimated METs (Fig. 2) tended to be approximately 200 kcal/day less than those reported by Ishikawa-Takata et al. [20, 21]. However, the maximum TEE was observed in participants in their 30s–40s, which is similar to results from previous studies [20, 21]. The TEE from the estimated METs was also approximately 100 kcal/day larger than the figure for sedentary adult women [22]. This clearly shows that the lifestyle of the nurses working in hospitals or clinics involves a high level of physical activity. These results also indicate that use of the JNHS questionnaire and estimated METs generate highly accurate estimates of the TEE.

**Table 3:** The total energy expenditure (TEE) of Japanese women

	Measurement	Age group	mean (SD) (years)	n	TEE
Ishikawa-Takata et al. [20]	doubly-labeled water method	20-29	25.3 (2.4)	8	1,954 kcal/d
		30-49	38.7 (4.4)	42	2,108 kcal/d
		50-69	62.0 (5.1)	49	2,039 kcal/d
Ishikawa-Takata et al. [21]	doubly-labeled water method	20-29	24.9 (2.7)	17	1,981 (361) kcal/d
		30-39	33.7 (2.8)	22	2,039 (394) kcal/d
		40-49	44.0 (3.0)	22	2,008 (234) kcal/d
		50-59	52.7 (2.0)	15	1,953 (220) kcal/d
Takata et al. [22]	human calorimeter	(seated adults)	32 (10)	20	1,772 (151) kcal/d

Using either estimated or midrange METs, time spent on MVPA significantly decreased with age (Fig. 3). With the estimated METs, the MVPA time significantly decreased from among those aged 20–29 to those in their 50s. The difference in MVPA time between those in their 50s and 60s was not significant, but the figure for the older group was lower. In contrast, when using the midrange METs, the MVPA time was not significantly different across the age groups.

A study of Japanese men and women aged 18–69 years showed that the time spent on MVPA had a negative correlation with age, regardless of sex [23]. Another study among Swedish women aged 56–75 years, found that the mean time spent on MVPA linearly decreased by 10 min/day with every 5 years of age [24]. This study’s results generated with the estimated METs are consistent with those previous findings. It is clear that it may be difficult to determine the physical activity level in individual participants with real accuracy. However, the physical activity question items included in the JNHS questionnaire appear to be sufficiently useful for epidemiological studies by using the estimated reference MET values.

With regard to the difference in the age-related change in the time spent in MVPA between using the estimated METs and the midrange METs, 2.4 METs for “moderate physical activity” in estimated METs was not included in MVPA, but 3.0 METs in midrange METs was included. Therefore, the MVPA hours per week using the estimated METs seem to track with age group as expected more so than using the midrange METs. With the midrange METs, the proportion of MVPA time for “moderate physical activity” increased with age, and the proportion of “heavy work” was higher among younger people (Table 2). The effects of age on the time spent on “heavy work” and “moderate physical activity” canceled each other out. The figures using the midrange METs therefore included more time spent on “moderate” and “vigorous” physical activity for participants in their 60s, who have shorter working hours. This age group therefore spent more time doing MVPA than participants in their 40s or 50s, who spend more working hours standing or walking. These results suggest that there might have been a type of reverse phenomenon because of increasing age.

This study had some limitations. First, Study 1 had a small sample size (*n* = 31). However, this was the same size as previous studies using an accelerometer for at least 7 consecutive days to validate new physical activity questionnaires, e.g. Lowther et al. [25], Mader et al. [26] and Meriwether et al. [27], who used sample sizes of 30, 35 and 41. Second, there might have been selection bias. Study 1 consisted of a sleep survey, a diet survey, and a survey of activities over 7 days. Participants needed to be interested in their own health and have time to spare. Consequently, Study 1 participants might have been health-conscious people who led healthy lifestyles. Third, the JNHS questionnaire was designed to be self-administered by female nurses, most of whom worked in hospitals. Applying the MET values estimated in this study to other Japanese women might therefore not be appropriate. If the same questions were used for a different population, suitable MET values would need to be investigated. However, despite the fact that this study included teaching staff and public health nurses, the result showing an age-associated change in the time spent on MVPA supports previous findings [23, 24]. This indicates the feasibility of general application of the JNHS questionnaire’s physical activity survey questions to other female populations as a simple physical activity survey.

## Conclusions

There are seven questions on physical activity in the JNHS questionnaire (four on work and three on physical activity outside work). These are all related to different physical activity intensities. By using the estimated reference MET values for physical activity intensities, this study has shown that the JNHS questionnaire is useful for epidemiological surveys.

## Data Availability

Data analyses are still in progress. In the future, the data base will be made available to other investigators.

## Abbreviations

BMR: Basal metabolic rate
BSA: Body surface area
CI: Confidence interval
DLW: Doubly-labeled water
EE: Energy expenditure
Ex: Exercise
GNHS: Gunma Nurses’ Health Study
ICC: Intra-class correlation coefficient
IPAQ: International Physical Activity Questionnaire
JNHS: Japan Nurses’ Health Study
MCMC: Markov Chain Monte Carlo
MET: Metabolic equivalent
MVPA: Moderate to vigorous intensity physical activity
SD: Standard deviation
TEE: Total energy expenditure
TEF: Thermic effect of food
